# A New Method to Obtain the Complete Genome Sequence of Multiple-Component Circular ssDNA Viruses by Transcriptome Analysis

**DOI:** 10.3389/fbioe.2020.00832

**Published:** 2020-07-21

**Authors:** Nai-tong Yu, Yu-liang Zhang, Jian-hua Wang, Zhi-xin Liu

**Affiliations:** ^1^Key Laboratory of Biology and Genetic Resources of Tropical Crops, Ministry of Agriculture and Rural Affairs, Institute of Tropical Bioscience and Biotechnology, Chinese Academy of Tropical Agricultural Sciences, Haikou, China; ^2^Hainan Key Laboratory of Tropical Microbe Resources, Haikou, China

**Keywords:** multi-component, DNA virus, nanovirids, transcriptomic sequencing, genome assembly

## Abstract

Circular single-stranded DNA (ssDNA) viruses are widely distributed globally, infecting diverse hosts ranging from bacteria, archaea, and eukaryotes. Among these, the genome of *Banana bunchy top virus* (BBTV) comprises at least six circular, ssDNA components that are ∼1 kb in length. Its genome is usually amplified and obtained at the DNA level. However, RNA-based techniques to obtain the genome sequence of such multi-component viruses have not been reported. In this study, transcriptome sequencing analysis showed that the full-length of BBTV each genomic component was transcribed into viral mRNA (vmRNA). Accordingly, the near-complete genome of BBTV B2 isolate was assembled using transcriptome sequencing data from virus-infected banana leaves. Assembly analysis of BBTV-derived reads indicated that the full-length sequences were obtained for DNA-R, DNA-U3, DNA-S, DNA-M, DNA-N, NewS2, and Sat4 components, while two gaps (73 and 25 nt) missing in the DNA-C component which was further filled by reverse transcription-PCR (RT-PCR). The RT-qPCR analysis indicated that the vmRNA levels of coding regions were 3.19–103.53 folds higher than those of non-coding regions, implying that the integrity of genome assembly depended on the transcription level of non-coding region. In conclusion, this study proposes a new approach to obtain the genome of nanovirids, and provides insights for studying the transcriptional mechanism of the family *Nanoviridae*, *Genomoviridae*, and *Geminiviridae*.

## Introduction

Circular single-stranded DNA (ssDNA) viruses are widely distributed worldwide, infecting diverse hosts from all three domains of bacteria, archaea, and eukaryotes (Lefkowitz et al., 2008). Currently, 11 families of ssDNA viruses have been established by the International Committee on Taxonomy of Viruses (ICTV, 2019 Release). These viruses are *Microviridae* and *Inoviridae* infecting bacteria, *Bacilladnaviridae* infecting diatoms, *Pleolipoviridae* and *Spiraviridae* infecting archaea, *Anelloviridae, Geminiviridae* and *Nanoviridae* infecting plants, *Circoviridae* infecting birds and mammals, *Genomoviridae* infecting plants and animals, and *Smacoviridae* that infects both animals and humans (Malathi and Dasgupta, 2019; Venkataraman and Selvarajan, 2019; Li et al., 2020). In addition to the aforementioned viruses, the satellite ssDNA molecules of nanovirids, and geminiviruses, which are not necessary for infection, have been reclassified into *Alphasatellitidae* and *Tolecusatellitidae* families (Zubair et al., 2017; Briddon et al., 2018).

Viruses that are members of the families *Nanoviridae*, *Genomoviridae*, and *Geminiviridae* have small genomes that replicate ssDNA using the rolling circle replication mechanism (Schmid et al., 2014; Weitzman and Fradet-Turcotte, 2018; Li et al., 2020). The family *Nanoviridae* comprises plant viruses that have very small virions with six to eight components, ranging in size from 1.0 to 1.1 kb; each component is separately encapsulated inside an individual virion (Yu et al., 2019b). *Banana bunchy top virus* (BBTV), belonging to the genus *Babuvirus* within family *Nanoviridae*, is known to infect all members of family *Musaceae* and is transmitted by aphids of the genus *Pentalonia* in a circulative manner (Watanabe et al., 2013, 2016; Kumar et al., 2015). The genome of BBTV contains six components, namely, DNA-R, DNA-U3, DNA-S, DNA-M, DNA-C, and DNA-N. Except DNA-U3, each component encodes a protein with known function. Some isolates may carry 1–3 distinct satellite DNA molecules (Yu et al., 2011). The replication initiator protein (Rep), also referred to as the master Rep encoded by DNA-R, is known to be solely necessary and sufficient for replicating all genomic components, whereas the replication initiator assistant protein (RepA) encoded by satellite DNA molecules cannot replicate any genomic components except its own DNA sequence (Venkataraman and Selvarajan, 2019). DNA-S encodes capsid protein (CP), which forms isometric particles, and aids in genome packaging (Ji et al., 2019). DNA-C, -N, and -M encode the cell-cycle link (Clink) protein, nuclear shuttle protein (NSP), and movement protein (MP), respectively, (Amin et al., 2011; Zhuang et al., 2019; Yu et al., 2019b). The function of DNA-U3 remains unknown for BBTV and other nanovirids. Rep and CP are evolutionarily more conserved than the other proteins and thus serve as useful markers for the identification and classification of ssDNA viruses (Yu et al., 2012). Furthermore, evolutionary analysis of concatenated amino acid sequences of Rep and CP is representative for ssDNA virus classification.

Considering the typical circular ssDNA of *Nanoviridae*, characteristic of multiple-component viruses, their complete genome can be directly obtained at the DNA level (Yu et al., 2012; Mukwa et al., 2016). In addition, rolling circle amplification (RCA) technology has also been applied to amplify the circular ssDNA genome in recent years (Jeske, 2018). However, these methods present some drawbacks for obtaining the viral genomes with two or more different isolates or reassortment components of the viral genome. Meanwhile, no RNA-based techniques and methods for obtaining the ssDNA virus genome have been reported so far. In this study, we explored a novel approach to obtain the complete genome of nanovirids based on transcribed vmRNA, which will extend the knowledge of transcriptional activity of the families *Nanoviridae*, *Genomoviridae*, and *Geminiviridae*.

## Materials and Methods

### Plant Samples

Samples of BBTV-infected banana leaves (B2) and healthy banana leaves (H4) were collected from Haikou, Hainan, China in October, 2013. Banana, the main planting line in Hainan province, is a species of *Musa* AAA Cavendish subgroup cv. Brazil. The BBTV-infected banana plant showed bunchy top with narrow and yellowing leaves. No symptoms were observed in the healthy banana sample. All banana leaves were collected and stored at −80°C in the laboratory.

### RNA Extraction, Library Preparation, and Transcriptome Sequencing

Total RNA extraction, cDNA library construction, and transcriptome sequencing of banana leaves were performed as described previously (Yu et al., 2019b). The high-quality of total RNA samples from banana leaves (CONC ≥ 108 ng/μL; OD_260__/__280_ = 2.01 ∼ 2.12; and RIN ≥ 6.6) were obtained and sequencing libraries were generated by using NEBNext^®^ Ultra^TM^ RNA Library Prep Kit. Accordingly, 100-bp paired-end reads were generated on an Illumina Hiseq 2000 platform. All downstream transcriptome analyses were based on high-quality clean data (Yu et al., 2019b).

### BBTV Reads Mapping and Genome Assembly

Reference BBTV genomes and gene model annotation files were downloaded from the National Center for Biotechnology Information (NCBI) website^1^. The dataset of downloaded BBTV genomes was indexed using Bowtie v2.0.6 and paired-end clean reads were aligned to the reference genomes using TopHat v2.0.9 (Kim et al., 2013). HTSeq v0.5.4p3 was used to count the number of viral reads that mapped to each component of the BBTV. RPKM values, Reads Per Kilobase of the exon model per Million mapped reads, a calculation of sequencing depth and gene length for read counts, were used for estimating gene expression levels as described (Mortazavi et al., 2008). In this study, the RPKM of BBTV each genomic component was calculated based on the length and reads numbers. The mapped BBTV reads were initially assembled into contigs in TopHat v2.0.9 and were further used to identify the possible BBTV genotypes in CodonCode Aligner (CodonCode, Centerville, MA).

### Complete Genomic Assembly of BBTV

Assembly analysis of the RNAseq reads, derived from the BBTV genome, indicated that the full-length of BBTV DNA-R, DNA-U3, DNA-S, DNA-M, DNA-N, NewS2, and Sat4 components were transcribed into viral mRNA (vmRNA), but not for the DNA-C component. To further reveal the full-length transcription of BBTV DNA-C component, the two primer pairs of DNA-C 61F/63R and DNA-C 903F/904R were designed based on the two gap regions (Supplementary Table 1). Following RT-PCR amplification, the fragments were cloned into the pMD19-T vector (Takara, Beijing, China), and then transferred into *Escherichia coli* DH5α competent cells (2nd Lab, Shanghai, China). Three positive clones were randomly selected and sent to Invitrogen (Guangdong, China) for Sanger sequencing.

Subsequently, the complete genome sequence of BBTV B2 was assembled from the RNA level. To further verify the accuracy of the assembled BBTV B2 genome, full-length nucleotide sequences of the eight genomic components of BBTV B2 were amplified using full-length specific primers (Supplementary Table 1). Total DNA of B2 and H4 was extracted by using DNAsecure Plant Kit (TIANGEN Biotech, Beijing). PCR amplification was performed as described previously (Yu et al., 2012).

### Phylogenetic Analysis of BBTV B2 Isolate

Respective amino acid (aa) sequences of Rep and CP from the Pacific-Indian Oceans group (PIO) and Southeast Asian group (SEA) were downloaded from the GenBank database in NCBI (Supplementary Table 2). The Rep and CP protein sequences of each BBTV isolate were concatenated sequentially, and multiple alignments of 14 sequences were performed using ClustalW in BioEdit (version 7.0.9.0). To study the relationship between BBTV B2 isolate and BBTV isolates from other parts of the world, a phylogenetic tree was constructed based on the concatenated amino acid sequences. Evolutionary analysis was conducted in MEGA6 and the evolutionary history was inferred using the Maximum Likelihood method based on the JTT matrix-based model (Tamura et al., 2013); *Abaca bunchy top virus* (ABTV) was used as an outgroup (Sharman et al., 2008).

### Verification of Full-Length Transcription of BBTV Genomic Components

To determine whether the full-length of BBTV genomic components was transcribed into vmRNA, RT-PCR was performed to identify the BBTV transcripts from the transcriptome sequencing data. In this study, the ORF vmRNA and vmRNA over the full-length of DNA-R, DNA-U3, DNA-S, DNA-M, DNA-C, DNA-N, NewS2, and Sat4 components were used to verify the BBTV genomic components transcription (Supplementary Table 1). The BBTV B2 cDNA (after removing DNA contamination) was used as a template, and PCR amplification was performed as described above. Total RNA and total DNA of BBTV B2 were used as negative and positive controls, respectively.

### Transcripts Levels Between Coding and Non-coding Regions of BBTV Genomic Components

Total RNAs of B2 leaves were extracted and subjected to cDNA synthesis as described above. To measure the transcription levels between coding and non-coding regions of BBTV genomic components, standard curves of each BBTV DNA component were constructed as Yu et al. (2019b). The eight standard plasmids were prepared previously (Yu et al., 2019a). Quantitative PCR (qPCR) was used to measure the vmRNA amounts between the coding and non-coding regions of BBTV genomic components including DNA-R, DNA-U3, DNA-S, DNA-M, DNA-C, DNA-N, NewS2, and Sat4. The qPCR was carried out using the SYBR green real-time PCR master mix reagents kit (CWBIO, China) on the StepOne real-time PCR system (Applied Biosystems, United States) with cycle conditions of 95°C for 5 min, followed by 40 cycles of 95°C for 15 s and 60°C for 1 min. The vmRNA level between coding and non-coding regions of BBTV genomic components in per μ *g* of banana leaf was measured three times independently with qPCR using the first stranded cDNAs as templates. Student’s *t*-test was used to evaluate the differences.

## Results

### BBTV Genome Assembly From Transcriptome Sequencing Data

The raw data of B2 and H4 are available in the NCBI database under accession number SRP129855 (Yu et al., 2019b). In total, 59.28 and 56.13 million raw reads were generated from B2 and H4 banana leaves, respectively, and then, 56.22 and 53.34 million clean reads were generated from these samples (Supplementary Table 3).

The genome of the BBTV B2 isolate was assembled from transcriptome sequencing data. In detail, contigs were assembled by mapping the reads onto the downloaded BBTV genomes, and these assembled contigs were used to search possible viruses and to remove banana genome sequences as much as possible. The clustering contigs were further used to assemble and identify the BBTV genotypes in CodonCode Aligner. The result showed that the full-lengths of DNA-R, DNA-U3, DNA-S, DNA-M, and DNA-N components of BBTV B2 were assembled from the BBTV transcribed reads, but not for the DNA-C component. Further analysis indicated that the sequences at 48–121 nt (gap1) and 814–839 nt (gap2) of DNA-C were not covered. Interestingly, the full-lengths of two satellite DNA components, designated as NewS2 and Sat4, were also obtained (Table 1).

**TABLE 1:** Reads coverage and BBTV genomic components of B2 sample.

**Isolate**	**Component**	**GenBank nos.**	**ORF (bp)**	**Read Counts**	**RPKM**
B2	DNA-R	MG545610	104–964	1022	42961.81
	DNA-U3	MG545611	143–409	4164	564461.46
	DNA-S	MG545612	227–742	8956	631875.55
	DNA-M	MG545613	282–32	1085	111881.29
	DNA-C	MG545614	239–724	44	3276.81
	DNA-N	MG545615	277–741	10953	852540.40
	NewS2	MG545617	62–919	1007	42628.32
	Sat4	MG545616	49–903	398	16789.22

**FIGURE 1:**
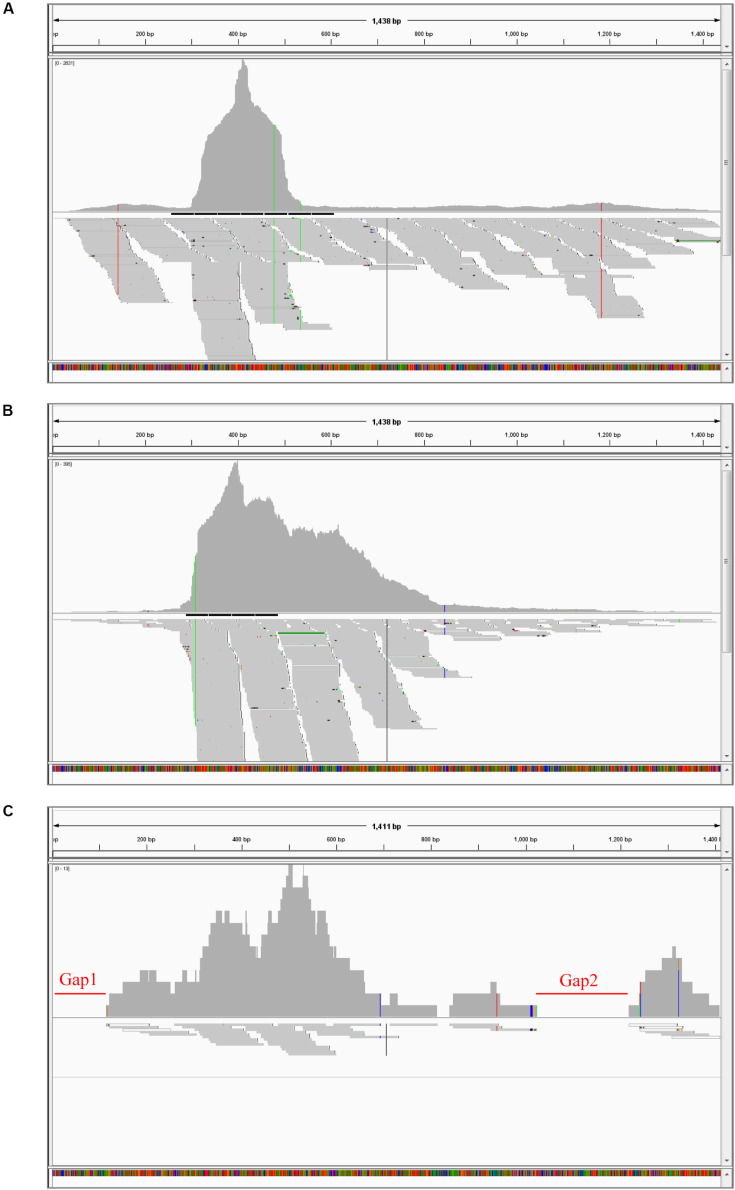
BBTV transcripts from the transcriptome sequencing data and mapping profiles of three representative components in BBTV B2 isolate. BBTV-derived reads and mapping profiles for DNA-U3 **(A)**, DNA-M **(B)**, and DNA-C **(C)**. The transcriptional reads were assembled by CodonCode Aligner version 3.7. Gap1 and gap2 represent nucleotide sequences with uncoverage regions.

To further clarify the BBTV assembly mechanism, the transcribed vmRNAs in the untranslated region (UTR) and the open reading frame (ORF) of three representative components (DNA-U3, DNA-M, and DNA-C) are analyzed in CodonCode Aligner. Briefly, a large number of vmRNA reads was transcribed from DNA-U3, DNA-M, and DNA-C ORFs, whereas a relatively low amount vmRNA reads was transcribed from their UTR regions, and some regions were not even covered, such as the sequences at 48–121 nt (gap1), and 814–839 nt (gap2) of the DNA-C (Figure 1). Therefore, except for the DNA-C component, these results showed that all DNA components from BBTV B2 were assembled, although the amounts of the transcribed reads of UTRs and ORFs were in different levels.

### Genomic Verification of BBTV B2

In order to fill the two gap regions of the DNA-C component of BBTV B2, two RT-PCR amplifications were conducted. Sequencing of three random clones of amplified fragment confirmed the assembled vmRNA sequences and the two gaps in the DNA-C component were filled. Subsequently, the complete genome sequence of BBTV B2 was obtained.

To further verify the accuracy of the BBTV B2 genome, the full-length nucleotide sequence of eight BBTV genomic components was amplified by regular PCR using Phusion High-Fidelity DNA Polymerase (NEB, United States), and the results confirmed the assembled vmRNA sequences of BBTV B2 genome. As shown in Table 1, there was only one BBTV genotype that was highly homologous to the BBTV Haikou isolate (Feng et al., 2010). Interestingly, its genome contained two satellite DNA components, NewS2, and Sat4. The assembled full-length nucleotide sequences of DNA-R (MG545610), DNA-U3 (MG545611), DNA-S (MG545612), DNA-M (MG545613), DNA-C (MG545614), DNA-N (MG545615), DNA-Sat4 (MG545616), and DNA-NewS2 (MG545617) from the BBTV B2 isolate were submitted to GenBank in NCBI.

### Phylogenetic Analysis of BBTV B2 Isolate

**FIGURE 2:**
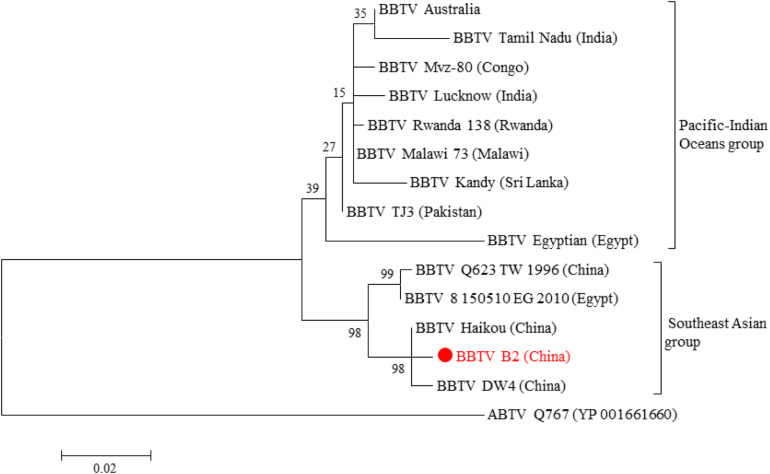
Phylogenetic tree based on the concatenated amino acid sequences of BBTV Rep and CP. Evolutionary analyses of BBTV B2 and BBTV isolates from other parts of the world were conducted in MEGA6. The evolutionary history was inferred by using the Maximum Likelihood method based on the JTT matrix-based model. The tree is drawn to scale, with branch lengths measured in the number of substitutions per site. The analysis involved 15 amino acid sequences (Supplementary Table 2). All positions containing gaps and missing data were eliminated. There were a total of 424 positions in the final dataset. ABTV was used as an outgroup.

Genetically, all BBTV isolates were clustered into two different groups, the SEA and PIO, confirming the BBTV classification proposed by Yu et al. (2012). The phylogenetic analysis showed that BBTV B2 was closer to other BBTV members isolated from China such as the isolates DW4 and Haikou (Figure 2). Meanwhile, clustering of the BBTV B2 isolate with the SEA group supports its evolution pattern. Interestingly, our analysis showed that the two isolates reported from Egypt, one (BBTV Egyptian) clustered with the PIO group whereas the other (BBTV 8150510EG2010) clustered with the SEA group. In all the cases, the ABTV isolate was an outgroup, as expected.

### Verification of the Full-Length Transcription of BBTV Genomic Components

According to the RPKM values determined from the transcriptome sequencing data, three components of DNA-N (852540.40), DNA-S (631875.55), and DNA-U3 (564461.46) were transcribed at high levels, followed by DNA-M component (111881.29) with moderate transcriptional level. The DNA-R component (42961.81) was transcribed at a low level, whereas the DNA-C component (3276.81) was even lower, more than 250-fold less than that of DNA-N component (Table 1). The two gaps represented the lowest amount of viral reads in the DNA-C component, but the reads of these regions was still transcribed and the vmRNA were further confirmed by the RT-PCR. The two satellite DNA components, NewS2 (42628.32), and Sat4 (16789.22), were transcribed at low levels as well.

**FIGURE 3:**
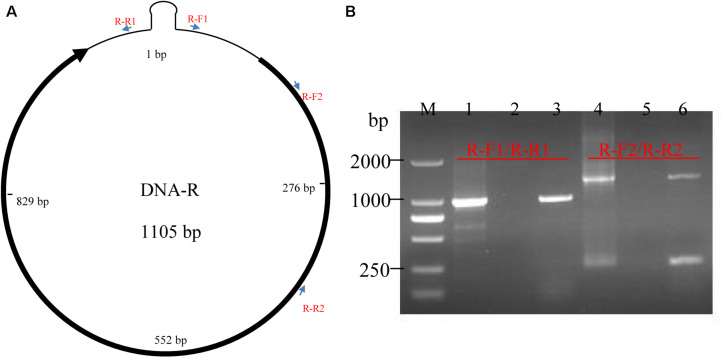
Detection of DNA-R full-length transcripts using RT-PCR. The schematic map of DNA-R component and primers localization **(A)**. Detection of DNA-R full-length transcripts using RT-PCR **(B)**. Lane M: DL2000 DNA marker; Amplification of DNA-R from total DNA (Lane 1), total RNA (Lane 2), cDNA (Lane 3) using R-F1/R-R1; and Amplification of DNA-R from total DNA (Lane 4), total RNA (Lane 5), and cDNA (Lane 6) using R-F2/R-R2.

To verify the full-length transcription of other BBTV genomic components, the RT-PCR was performed on the vmRNA of DNA-R, DNA-U3, DNA-S, DNA-M, DNA-N, NewS2, and Sat4 components. The result showed that expected DNA bands (1.0–1.1 kb) were obtained, indicating the full-length transcription of these DNA components. In addition, two primer-pairs targeting the coding and non-coding region of DNA-R were designed (Figure 3A). The RT-PCR showed that a specific band containing the DNA-R ORF was successfully amplified by the R-F1/R1 primers, whereas two bands were amplified by the R-F2/R2 primers. To confirm the two bands amplified by R-F2/R2, the fragments were subjected to Sanger sequencing and the results indicated that the smaller DNA band was about 300 bp whereas the large band was ∼1400 bp, which was over the full-length of the DNA-R component (Figure 3B). As controls, the PCR obtained from the total DNA sample was positive, whereas a negative result was obtained in the RNA sample, indicating the RNA was not contaminated with DNA. These results indicated that BBTV genomic components can be transcribed into vmRNA with over the full-length (one copy) of their circle DNA molecules, which can be further used to obtain the BBTV genome by transcriptome sequencing.

### Transcriptional Levels of Coding and Non-coding Regions of Each BBTV Genomic Component

**FIGURE 4:**
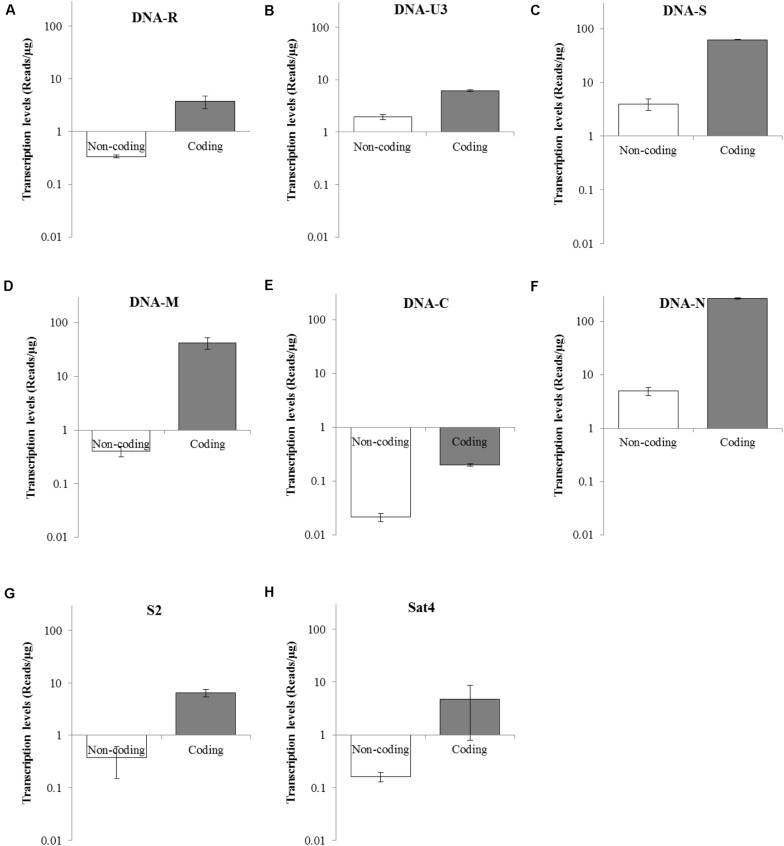
Transcriptional levels of coding and non-coding regions of BBTV each component. The transcriptional levels of non-coding and coding regions of DNA-R **(A)**, DNA-U3 **(B)**, DNA-S **(C)**, DNA-M **(D)**, DNA-C **(E)**, DNA-N **(F)**, NewS2 **(G)**, and Sat4 **(H)** by RT-qPCR analysis. Student’s *t*-test was used to evaluate the differences.

To determine the transcriptional levels of the coding and non-coding regions of each BBTV genomic component, primer-pairs targeting specific regions were used for RT-qPCR (Table 2). In detail, the coding region of DNA-N was highly transcribed with reads number of 268.50 copies/μg, followed by the medium level of DNA-S (62.67 copies/μg), and DNA-M (42.24 copies/μg), whereas DNA-C was the lowest transcribed region (0.2 copies/μg). For non-coding regions, DNA-N (4.99 copies/μg), and DNA-S (3.99 copies/μg) were transcribed at medium-low levels, but DNA-C had the lowest value of reads number at 0.02 copies/μg (Figure 4). Accordingly, the relative transcriptional level of coding region was 3.19–103.53 fold higher than that of non-coding region for each BBTV component. Therefore, the complete genome of BBTV assembly depended on the vmRNA content of non-coding regions, especially for the DNA-C component. This further verified the full-length transcription of BBTV genomic components.

**TABLE 2:** Primers used for RT-qPCR in this study.

**Primer name**	**Sequences (5′–3′)**	**Usage**
DNA-R ncF	GGGAAAAGCAAAGATTCGGG	DNA-R
DNA-R ncR	AGTGTACGATTGGCATAGCG	RT-qPCR
DNA-R cF	GCACACCTTGAGAAACGAAAG	
DNA-R cR	TCAACTCTGCTTGCACTCTG	
DNA-U3 ncF	CCCCTAACTAGCGTGATGTATG	DNA-U3
DNA-U3 ncR	GACCATCTACCTTGACCTTCG	RT-qPCR
DNA-U3 cF	GCTCTCGCTCTTCTGTCAAAG	
DNA-U3 cR	AATACCCGATATACGCTTCGC	
DNA-S ncF	CATATGTCCCGAGTTAGTGCG	DNA-S
DNA-S ncR	GCCCAAAACCCATCTTAACG	RT-qPCR
DNA-S cF	AGGAAGTATGGAAGCAAGGC	
DNA-S cR	TGTAGTCGGCTGGTTGATTTC	
DNA-M ncF	AGTTAGTGCGCCACGTAAG	DNA-M
DNA-M ncR	GATACGATAGACCAATCAACCCC	RT-qPCR
DNA-M cF	CAGAGCGGGTGAAACAATTC	
DNA-M cR	TTCTGCATCCATACACGTCG	
DNA-C ncF	GGCCCGTTTAAATATGTGTTGG	DNA-C
DNA-C ncR	CACTAACTCGGGACATGGAC	RT-qPCR
DNA-C cF	TGGAATTCTGGGAATCGTCTG	
DNA-C cR	ATCCTTCTGACACAGCACTTC	
DNA-N ncF	CGGGACATGACGTAAGCATAG	DNA-N
DNA-N ncR	TTTATTCGGCAGAGACAGGC	RT-qPCR
DNA-N cF	TGGCTGTGATTGGAAGACG	
DNA-N cR	AGTACTGGACGCATTGTTCC	
NewS2 ncF	CCTTACACCTCTGCCTTACAC	NewS2
NewS2 ncR	TGCCACCGACATCCTTATTG	RT-qPCR
NewS2 cF	TGGCGTCCTCTAAATGGTG	
NewS2 cR	ATTCTGTTCGTCTGTTCCTCTC	
Sat4 ncF	CAACACCTTTAACCTCTGCG	Sat4
Sat4 ncR	TCCCCTTTTGTCATAGCGTAC	RT-qPCR
Sat4 cF	TGGCAGGTACAATTAACGGAG	
Sat4 cR	CTCCCCTTGTGTAGAACCAATC	

## Discussion

The genomes of most ssDNA viruses, particularly nanovirids, genomoviruses, and geminiviruses, are obtained using DNA-based methods such as PCR and RCA (Bashir et al., 2012; Yu et al., 2012). In recent years, high-throughput DNA sequencing technology has also been successfully applied to obtain the ssDNA virus genome (Dayaram et al., 2015; Gallet et al., 2017). However, RNA-based techniques to obtain the genome sequence of such multi-component viruses have not been reported. In this study, we explored a novel approach to obtain the complete genome of BBTV, a multiple-component circular ssDNA virus. The full-lengths of DNA-R, DNA-U3, DNA-S, DNA-M, DNA-N, NewS2, and Sat4 components of BBTV B2 were assembled from the Illumina reads, but the *de novo* assembly from the BBTV transcribed reads failed to obtain the full-length nucleotide sequences of DNA-C component. This is most likely due to the rare viral reads mapped onto the gapped regions. Therefore, the BBTV transcribed reads from the transcriptome sequencing data would not completely cover these regions. Similar results were observed in potyvirus, enamovirus, and nucleorhabdovirus (Cao et al., 2019; Yu et al., 2020). Considering the cost involved, we chose to fill the gap regions by RT-PCR. However, these results also indicated the whole genome of BBTV would likely be obtained by increasing the transcriptomic sequencing data coverage. To the best of our knowledge, this study is the first report using the transcriptome sequencing data to assemble the whole genome of BBTV.

Complete genome assembly using BBTV RNA reads revealed that a large amount of vmRNA in the coding region was transcribed in each component, whereas a relatively low amount of vmRNA was transcribed in the non-coding region (UTR). Although the assembled DNA-C component had two small gaps from the Illumina reads, RT-PCR was conducted to fill the gap regions and allowed to determine the full-length of DNA-C, implying that the integrity of the genome assembly depended on the transcriptional levels of non-coding regions, especially for the DNA-C component. The RT-qPCR further confirmed this result. Sequence analysis revealed that the poly(A) signal sequence was found downstream of the ORFs in each component, but the vmRNA from the UTR was still transcribed. This may be due to the incompletely terminated transcriptional regulation of BBTV and it is very common that the partial sequence of 3’ UTR transcribes (Hilgers, 2015; Zhang et al., 2015). Based on this transcriptional feature, the whole genome of BBTV might be obtained by transcriptome high-throughput sequencing with sufficient deep-sequencing data.

For multi-component ssDNA viruses, there are some drawbacks to obtain the viral genomes by using the conventional PCR-based or RCA methods. Firstly, the plant may be infected with two or more different viral isolates, and thus, when using PCR amplification, primers may bind to non-predominant target sequences and amplify the non-dominant viral isolates. Secondly, as a multi-component virus, the obtained genome sequences may not represent an isolate or the sequence may come from different isolates. Thirdly, the inter-reassortment component from two BBTV isolates or intra-reassortment component in an isolate could not be recognized (Grigoras et al., 2014; Kraberger et al., 2018). Lastly, some BBTV isolates may contain 1–3 distinct satellite molecules. The nucleotide sequences of these satellite components have large differences, and degenerate primers often fail to obtain all the satellite molecules. However, the aforementioned disadvantages can be overcome using transcriptome high-throughput sequencing. In this study, there was apparently only one BBTV genotype, BBTV B2, excluding the possibility of contamination by different isolates in the infection. We also identified a new satellite molecule, NewS2, which has not been reported yet.

In conclusion, transcriptome sequencing analysis showed that the full-length of BBTV genomic components was transcribed into vmRNA. Based on this characteristic, its complete genome could be assembled and the obtained sequences were identical to the DNA method. This study found the new characteristic of a full-length transcription rather than one copy of BBTV each component and provides a new approach to obtain the complete genome of BBTV, which will extend the knowledge of transcriptional mechanism of the nanovirids, genomoviruses and geminiviruses.

## Data Availability Statement

The datasets generated for this study can be found in the GenBank MG545610–MG545617.

## Ethics Statement

The authors declare that ethical standards have been followed and that no human participants or animals were involved in this research.

## Author Contributions

NY conceived and designed the research. YZ and JW collected the samples. NY and ZL analyzed and wrote the data. All authors contributed to the article and approved the submitted version.

## Conflict of Interest

The authors declare that the research was conducted in the absence of any commercial or financial relationships that could be construed as a potential conflict of interest.

## Footnotes

1 https://www.ncbi.nlm.nih.gov/nuccore/?term=banana%20bunchy%20top%20virus
