# Supplementary material for: A New Method to Obtain the Complete Genome Sequence of Multiple-Component Circular ssDNA Viruses by Transcriptome Analysis
Source: Front Bioeng Biotechnol. 2020 Jul 21;8:832. doi: 10.3389/fbioe.2020.00832 (PMC7396673; doi:10.3389/fbioe.2020.00832)
Supplement: Supplementary file 1 [file Table_1.docx]

**Supplementary Table 1.** Primers used in this study.

| **Primer name** | **Sequences (5’-3’)** | **Usage** |
| --- | --- | --- |
| DNA-R F159 | GAAGTGAAGGCGGATTGTTGATG | DNA-R |
| DNA-R R158 | TCCAGTGATGCGGGATGAGTT |  |
| DNA-U3 F841 | GCAATCAAATCTAACCGTTCATCC | DNA-U3 |
| DNA-U3 R841 | CTTAGCCACGAAGGAAGGAATCT |  |
| DNA-S F995 | GGCACAACTACACCTTCCTTCTC | DNA-S |
| DNA-S R995 | CGGATAAGGATGAGAACCACC |  |
| DNA-M F320 | TTCAAAGAATAGTTTCACCCGC | DNA-M |
| DNA-M R319 | AATGGTTTCTGTTAGTTGGAGCA |  |
| DNA-C F563 | GATATACCGAGTAGTCACCACC | DNA-C |
| DNA-C R563 | CAAGAGTTGTATTGTGATGAGG |  |
| DNA-N F269 | GCTTCGCATACGCTCTGATTTA | DNA-N |
| DNA-N R266 | AAGCAGAAGCGATGGATTGGG |  |
| NewS2 F987 | CGTGTTATCGTAGTGGTGGGGTCC | NewS2 |
| NewS2 R985 | ACGCTATGCCGTACAACCAAGTC |  |
| Sat4 F304 | ACGCCTTGTTGTCGTAATCTG | Sat4 |
| Sat4 R302 | CGTACTGTTCTAATGAAGCCCTA |  |
| DNA-C 61F | ATGCACGTGAGAAGGCAGTT | DNA-C gap |
| DNA-C 63R | CATTTGTTAAATGCACGTGATGTCC |  |
| DNA-C 903F | GCCCGTTTAAATATGTGTTGGACG | DNA-C gap |
| DNA-C 904R | CCTTCGGCCCTTAATCGTTAAG |  |
| R-F1 | ATAAATAGACCTCCCCCCC | DNA-R RT-PCR |
| R-R1 | ATAGCACAATCAACCCCCG |  |
| R-F2 | ACTTCCAGCGATGCGGGATG | DNA-R RT-PCR |
| R-R2 | AAGCACCAAACTCGAAGGGAC |  |
