# Supplementary material for: A New Method to Obtain the Complete Genome Sequence of Multiple-Component Circular ssDNA Viruses by Transcriptome Analysis
Source: Front Bioeng Biotechnol. 2020 Jul 21;8:832. doi: 10.3389/fbioe.2020.00832 (PMC7396673; doi:10.3389/fbioe.2020.00832)
Supplement: Supplementary file 2 [file Table_2.docx]

**Supplementary Table S2.** List of Rep and CP amino acids in different BBTV isolates used in this study.

| **Protein** | **Accession no** | **Place of origin** | **Isolate** |
| --- | --- | --- | --- |
| BBTV Rep | ACK43789 | Haikou,China | Haikou |
| BBTV Rep | AZL93958 | Haikou,China | B2 |
| BBTV Rep | ALU11293 | Guangdong, China | DW4 |
| BBTV Rep | AKO71391 | Taiwan, China | Q623_TW_1996 |
| BBTV Rep | AOC83974 | Congo | Mvz-80 |
| BBTV Rep | AKO71319 | Egypt | 8_150510_EG_2010 |
| BBTV Rep | AFN27070 | Rwanda | Rwanda 138 |
| BBTV Rep | AFN27075 | Malawi | Malawi 73 |
| BBTV Rep | ABB59944 | India | Lucknow |
| BBTV Rep | AGF92158 | Pakistan | TJ3 |
| BBTV Rep | AEV53366 | Sri Lanka | Kandy |
| BBTV Rep | NP_604483 | Australia | Australia |
| BBTV Rep | ACA34591 | India | Tamil Nadu |
| BBTV Rep | AAC98788 | Egypt | gyptian |
| ABTV Rep | YP_001661660 | Malaysia | Q767 |
| BBTV CP | ACK43790 | Haikou,China | Haikou |
| BBTV CP | AZL93959 | Haikou,China | B2 |
| BBTV CP | ASV46566 | Guangdong, China | DW4 |
| BBTV CP | AKO71247 | Taiwan, China | Q623_TW_1996 |
| BBTV CP | AON96281 | Congo | Mvz-80 |
| BBTV CP | AKO71177 | Egypt | 8_150510_EG_2010 |
| BBTV CP | AFN27071 | Rwanda | Rwanda 138 |
| BBTV CP | AFN27076 | Malawi | Malawi 73 |
| BBTV CP | ABS12247 | India | Lucknow |
| BBTV CP | AGF92159 | Pakistan | TJ3 |
| BBTV CP | AEV53368 | Sri Lanka | Kandy |
| BBTV CP | AAA87368 | Australia | Australia |
| BBTV CP | ACF24825 | India | Tamil Nadu |
| BBTV CP | AAC98792 | Egypt | gyptian |
| ABTV CP | YP_001661657 | Malaysia | Q767 |
