# Supplementary material for: A New Method to Obtain the Complete Genome Sequence of Multiple-Component Circular ssDNA Viruses by Transcriptome Analysis
Source: Front Bioeng Biotechnol. 2020 Jul 21;8:832. doi: 10.3389/fbioe.2020.00832 (PMC7396673; doi:10.3389/fbioe.2020.00832)
Supplement: Supplementary file 3 [file Table_3.docx]

**Supplementary Table S3.** Summary statistics of two transcriptome sequencing data and quality assessment (Yu et al., 2019b).

| **Sample** | **Raw reads** | **Clean reads** | **clean bases** | **Error rate (%)** | **Q20 (%)** | **Q30 (%)** | **GC content (%)** |
| --- | --- | --- | --- | --- | --- | --- | --- |
| B2_1 | 29637569 | 28112101 | 2.81G | 0.04 | 97.13 | 91.48 | 50.36 |
| B2_2 | 29637569 | 28112101 | 2.81G | 0.04 | 96.42 | 90.35 | 50.40 |
| H4_1 | 28063391 | 26670630 | 2.67G | 0.04 | 97.00 | 91.19 | 50.88 |
| H4_2 | 28063391 | 26670630 | 2.67G | 0.04 | 96.50 | 90.48 | 50.92 |

Q20 (%): the percentage of sequences with a sequencing error rate lower than 1%.
